# Hand Rejuvenation by Autologous Fat Grafting in Post-Hansen's Hand Atrophy: Aesthetic and Psychological Implications

**DOI:** 10.1055/s-0044-1791943

**Published:** 2024-11-04

**Authors:** Venkata Ramana Yamani, Swamy Vivek Gurindagunta, Rama Linga Raju, Shree Kumar, Mukesh Krishna Valluri, Mrigya Sharma

**Affiliations:** 1 Department of Plastic Surgery, Contours Plastic and Aesthetic Clinic, Hyderabad, Telangana, India; 2 Department of Anaesthesia, Consultant, Contours Plastic and Aesthetic Clinic, Hyderabad, Telangana, India; 3 Department of Plastic Surgery, Contours Plastic and Aesthetic Clinic, Medical College, Gotri, Gujarat, India

**Keywords:** autologous fat grafting, Hansen's disease, microfat, hand aesthetics

## Abstract

**Introduction** Contemporary medical science has been using fat grafting in aesthetic and reconstructive procedures, consistently achieving successful outcomes. Hansen's disease, caused by *Mycobacterium leprae* , leads to hand deformities due to peripheral neuropathy, resulting in soft-tissue atrophy, volume loss, and compromised hand function. Tendon transfer surgery is a common remedy for functionality, but it often does not address aesthetic concerns and the patient's psychological impact of living with an atrophic hand. Autologous fat grafting can effectively address these concerns.

**Materials and Methods** This prospective study evaluates the efficacy of fat grafting for hand rejuvenation in patients with Hansen's disease posttendon transfer surgery, focusing on aesthetic and psychological outcomes. We recorded data from 12 patients who underwent the procedure between 2015 and 2024. Using the Coleman technique, fat was harvested from the paraumbilical region of the abdomen and injected into various hand regions.

**Results** Autologous fat grafting showed high patient satisfaction with significant improvements in hand contour, skin texture, and psychological health. The benefits of the procedure included improved self-esteem, enhanced quality of life, reduced social stigma, and psychological well-being.

**Conclusion** Autologous fat grafting is a safe and effective technique for hand rejuvenation in patients with Hansen's disease, after functional treatment addressing both physical deformities and their psychological impacts. It could be considered one of the components in the comprehensive management of Hansen's disease–related hand deformities, significantly enhancing patients' overall quality of life.

## Introduction

Since the dawn of human civilization, the visual appeal of the hands has commanded significant attention and fascination, representing the elevated status of mankind and serving as a direct expression of human ingenuity. Within the sphere of artistic expression, the intricate movements of the hands have been acknowledged as a tangible and expressive form of communication, which, despite displaying discernible variances, has been standardized over successive centuries. In the realm of contemporary medical science, autologous fat grafting has remained a long-standing practice in both aesthetic and reconstructive surgery, consistently delivering favorable outcomes. 1 2

The history of using fat grafting to correct concavities goes back over a century. 3 In 1988, the technique of autologous fat grafting for hand rejuvenation was first recorded, which involved depositing a fat bolus in the proximal dorsum of the hand and then massaging to achieve the desired contour. 4 This was followed by innovative strategies such as combining lipofilling with laser resurfacing in 1989 and introducing hand rejuvenation with microlipoinjection for age-related cosmetic improvement in 1990. 5 The first published patient series, reporting excellent patient satisfaction (98.6%), was released in 1992. 6 In 2002, Coleman published a significant study detailing a method for structural fat grafting, which involved making multiple passes with depositions of small quantities to produce more consistent results compared to injecting a lump of fat and manipulating it throughout the hand. 7

Hansen's disease, attributed to *Mycobacterium leprae* , is a chronic infectious condition primarily affecting the skin and peripheral nerves. Among the significant complications of Hansen's disease are the resultant hand deformities stemming from peripheral neuropathy, leading to soft-tissue atrophy, loss of volume, contractures, and altered sensation. 8 These deformities not only adversely affect aesthetics but also impede hand function and diminish the patient's overall quality of life.

Tendon transfer surgery is a commonly employed procedure aimed at restoring hand function in individuals affected by Hansen's disease. 9 Despite the successful restoration of function, hand aesthetics may be compromised, leading to psychological distress. 10

Fat grafting has emerged as a technique for aesthetic enhancement, improving tissue quality, and delivering long-term results like reduction of ulceration risk, softening of contracted areas and reduced fibrosis. 11 Fat grafting entails the extraction of fat from one part of the body, its processing, and subsequent injection into the target area. The adipose tissue serves as a filler, reinstating volume and improving tissue quality.

The purpose of this study was to evaluate the aesthetic and psychological benefits of fat grafting in patients with Hansen's disease with hand atrophy who have undergone tendon transfer surgery by analyzing both clinical outcomes and patient-reported experiences.

## Objective

This prospective study aims to evaluate the efficacy of fat grafting for hand rejuvenation in posttendon transfer Hansen's disease patients and its aesthetic and psychological implications on them.

## Materials and Methods

We conducted a prospective analysis of 12 patients with Hansen's disease who underwent fat grafting for hand rejuvenation between 2015 and 2024. All the data were collected on patient demographics, disease characteristics, patient's past and surgical history, surgical technique implemented, complications, and outcomes. Subjective analysis for psychological assessment was done using Center for Epidemiologic Studies Depression (CES-D) 12 scale for assessing depression and Generalized Anxiety Disorder 7 (GAD-7) 13 scale for assessing anxiety.

The CES-D scale is a self-reported depression scale designed to measure depressive symptoms in the general population. The CES-D scale consists of 20 items, each rated on a 4-point scale, with the following response options: 0—rarely or none of the time (<1 day); 1—some or a little of the time (1–2 days); 2—occasionally or a moderate amount of time (3–4 days); and 3—most or all of the time (5–7 days).

GAD-7 is a self-reported questionnaire designed to identify whether a person may have generalized anxiety disorder and to assess the severity of their anxiety symptoms. The GAD-7 consists of seven questions, with each question scored from 0 to 3. The total score ranges from 0 to 21, with higher scores indicating more severe anxiety symptoms.

It is unusual for patients with Hansen's disease to show up for cosmetic corrections, but whatever cases we had done were referred to us by a physical therapist who works at a leprosy rehabilitation center and helps with the posttendon transfer exercises to cure patients with Hansen's disease predominantly. Out of all cases the physical therapist encountered, only a few young people who had cosmetic concerns were educated and were referred to us.

### Inclusion Criteria

- All patients tested negative for *M. leprae* infection.
- All patients who completed their course of treatment, including medical management, tendon transfers, physical therapy, and rehabilitation, achieving acceptable hand function.
- All patients who exhibited post-Hansen's disease atrophy of the hands.

**Surgical technique:** Both general anesthesia and local anesthesia were utilized, depending on the case.

**Donor site:** The paraumbilical site in the abdomen was used as the donor site in all cases.

### Procedure



**Video 1** Video demonstrating the procedure of autologous fat grafting in Post-Hansen–s hand atrophy.

**Fig. 1:**
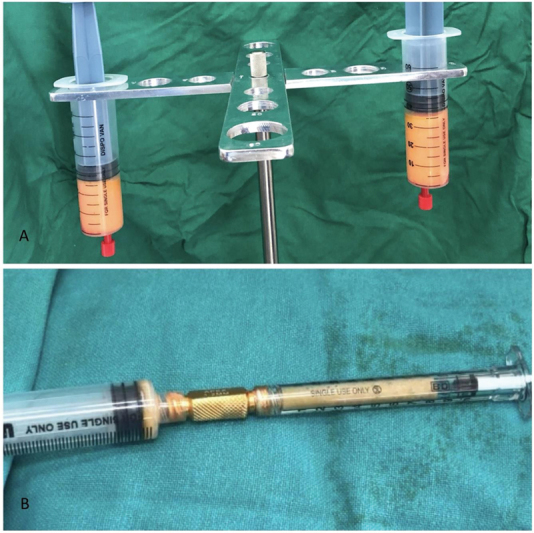
( **A** ) Centrifugation of the fat after harvest. ( **B** ) Conversion of autologous fat into microfat for injecting.

**Fig. 2:**
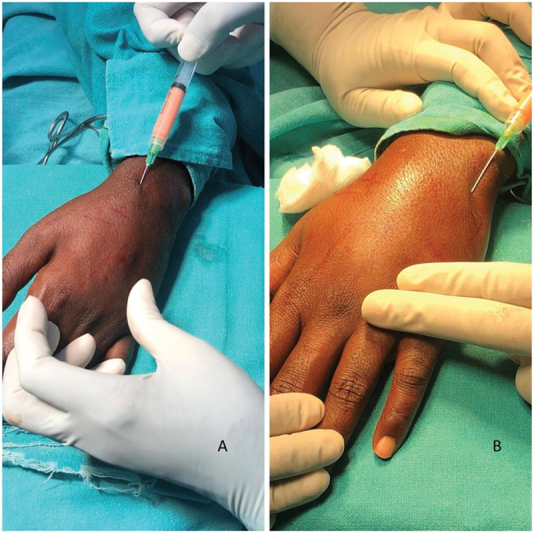
( **A, B** ) Process of injection of microfat into the premarked areas on the dorsum of the hand using a blunt-tip 26-gauge cannula.

- Syringe liposuction was performed using a 2.5-mm cannula and a 20-mL Luer lock syringe.
- The harvested fat was centrifuged ( Fig. 1A ) and filtered ( Fig. 1B ) to produce microfat.
- Fat was injected using a 1-mL BD Luer lock syringe with a 26-gauge cannula into the dorsum of the hand, uniformly in subcutaneous plane, paratenon atrophic gutters, the first web space, digital skin (subcutaneously), interphalangeal (IP) joints, and thenar area ( Fig. 2 ; Video 1 ).
- An average volume of 50 to 60 mL of fat was injected into each hand, with a slight overcorrection.

### Postoperative Care

- A cotton crepe bandage dressing was applied immediately after the procedure.
- Hand elevation was maintained for 1 week.

**Assessment:** Evaluations were conducted at 1 week, 4 weeks, and 3 months postoperatively. Subjective assessments of the results were performed, accompanied by photographic comparisons to document outcomes. Psychological improvement was observed through subjective history.

## Results

Out of the 12 patients included in the study, 10 were males and 2 were females, with a mean age of 25 years. All the patients were unmarried before the surgery. They completed basic education. The average volume of fat injected into each patient was 50 to 60 mL. Approximately 10 to 15 mL overcorrection was done in all the patients in view of expected atrophy. Only one patient turned up for repeat fat grafting.

Fat grafting was performed using the Coleman technique, which involves careful harvesting, processing, and reinjection of fat to ensure maximal survival and integration of the graft. The mean volume of fat injected was 50 to 60 mL per hand.

**Fig. 3:**
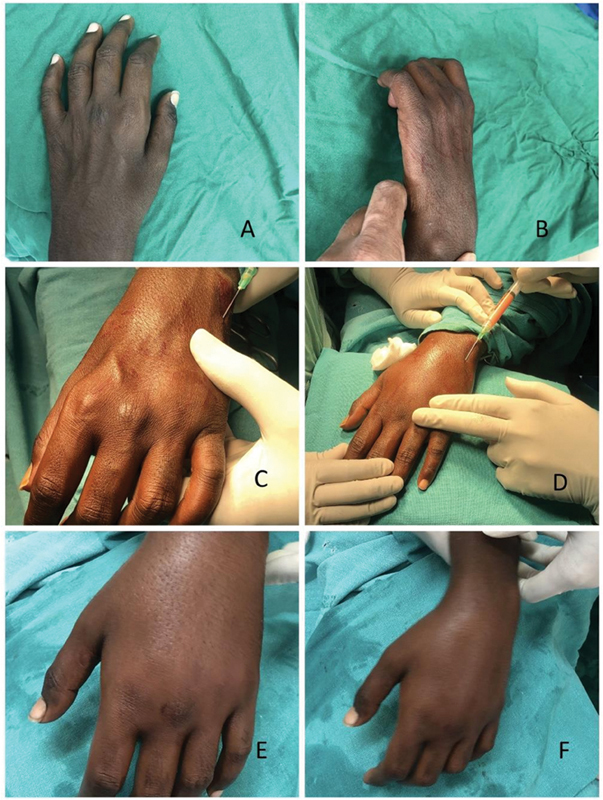
Case 1. ( **A, B** ) Preoperative pictures showing the atrophic hand. ( **C, D** ) Intraoperative pictures during the process of injection. ( **E, F** ) Immediate postoperative pictures showing an improved appearance.

Patient satisfaction was high, and all the patients noticed a significant improvement in hand contour and skin texture improvements postoperatively ( Figs. 3 4 5 ).

**Fig. 4:**
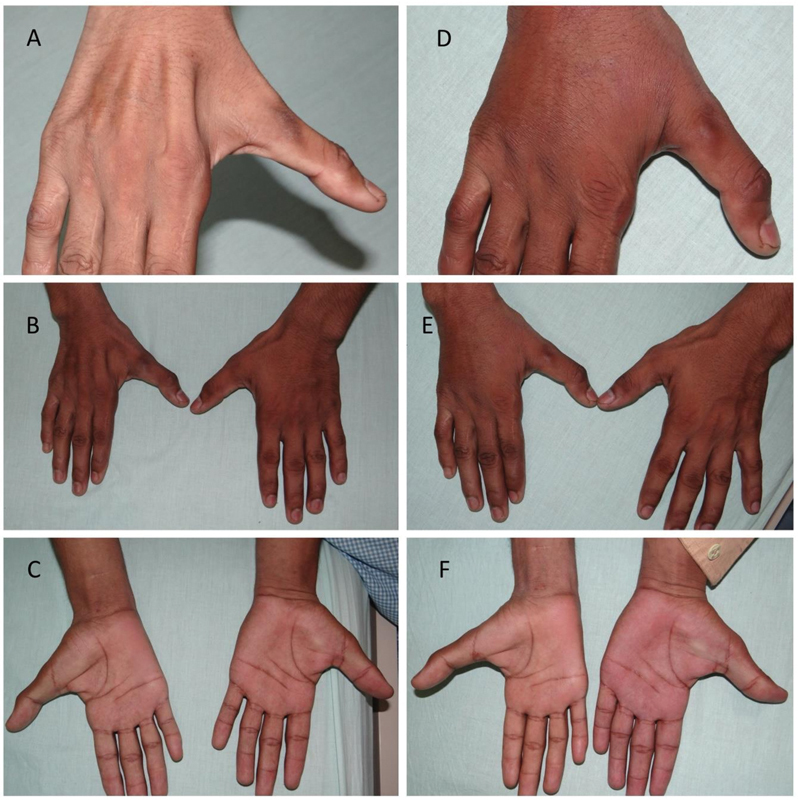
Case 2. ( **A–C** ) Pictures showing the preoperative hands. ( **D–F** ) Pictures showing postoperative results after 3 months.

**Fig. 5:**
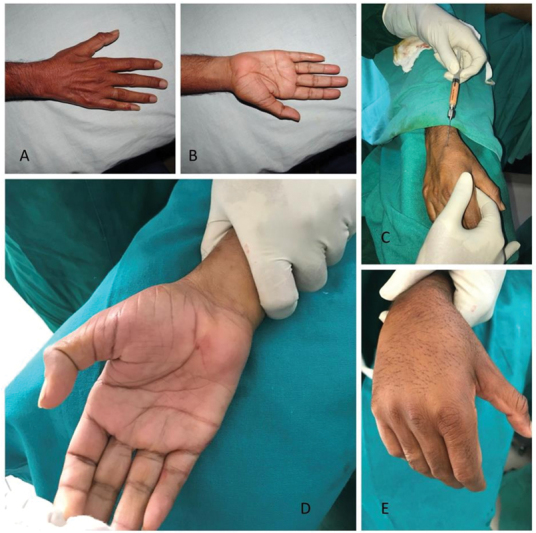
Case 3. ( **A, B** ) Pictures showing preoperative atrophic hand. Note the atrophic paratendon gutters. ( **C** ) Intraoperative injection of the autologous fat. ( **D, E** ) Immediate on-table result of the patient with good filling and disappearance of gutters.

All the patients were assessed for complications resulting from the fat grafting procedure.

In one case, infection was noticed at 72 hours postoperatively. Multiple small abscesses were observed, for which incision and drainage were done. On culture sensitivity testing, atypical *Mycobacterium* was reported, with sensitivity to clarithromycin. Dressings were done twice weekly. Injectable antibiotics were given for 2 weeks and oral antibiotics for another 2 weeks.

**Fig. 6:**
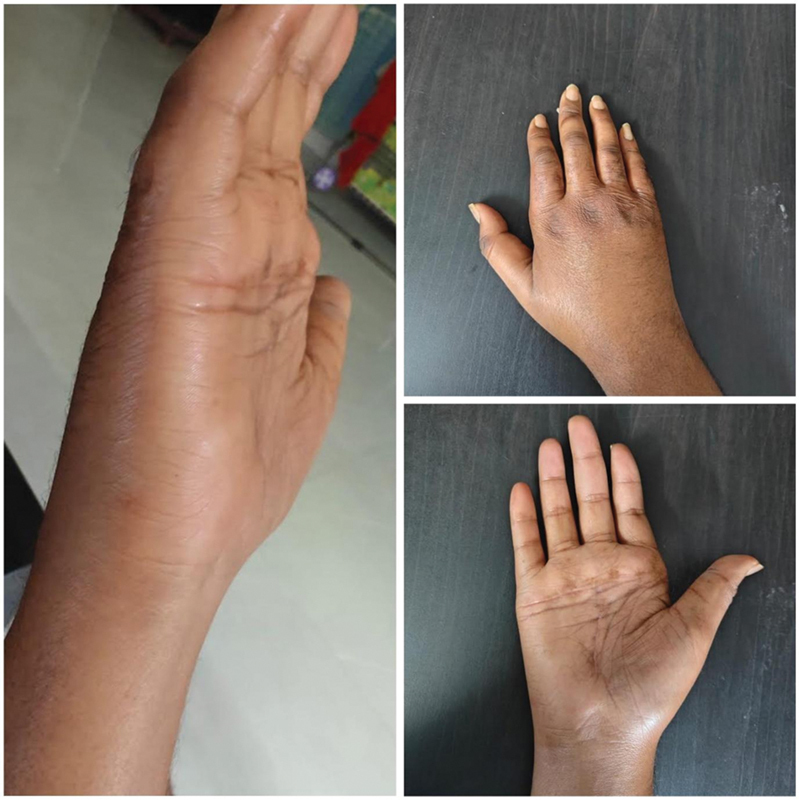
Pictures showing the long-term result after 7 months. The filling is maintained and the patient is satisfied.

**Fig. 7:**
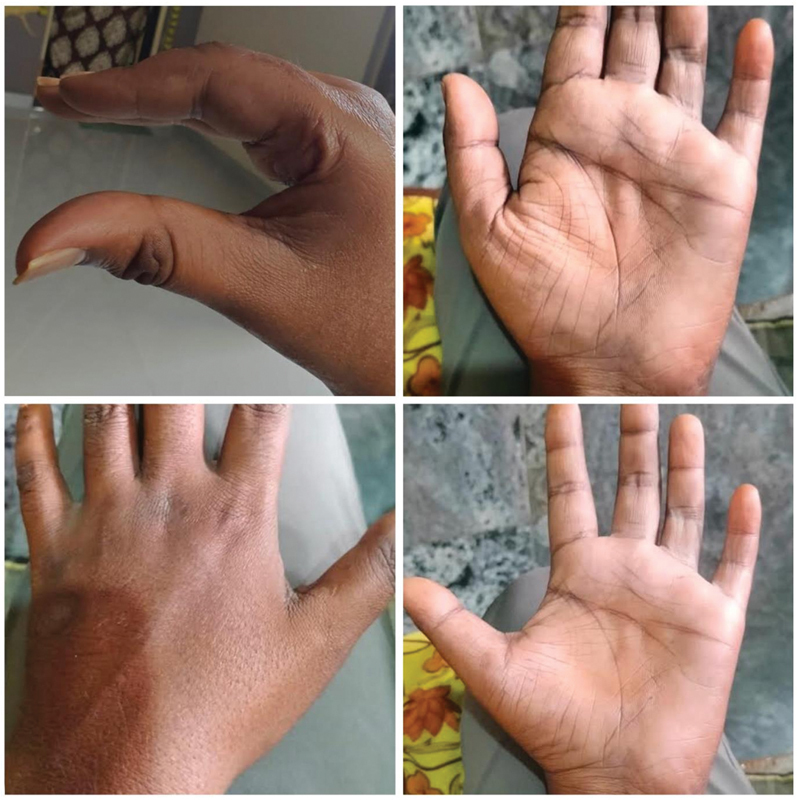
The 2-year long-term follow-up photographs of a patient with satisfactory results.

No other major complications were reported in other cases. Minor issues like swelling and bruising were noted but resolved within a few weeks. All patients reported improved hand aesthetics on long-term follow-up ( Figs. 6 and 7 ).

Repeat procedure was done in only one case after 3 months on the patient's request.

The positive outcomes support the use of fat grafting for hand rejuvenation in patients with Hansen's disease.

Psychological evaluation is done using the CES-D scale for assessing depression and GAD-7 for assessing anxiety levels preoperatively and after 3 months of follow-up.

Our study demonstrated that fat grafting in hand rejuvenation posttendon transfer significantly impacts patients, reducing depression levels as indicated by the CES-D scores. The mean CES-D scores decreased from 20.08 (moderate depressive symptoms) to 9.83 (minimal depressive symptoms) postintervention, highlighting the potential benefits of this procedure for improving psychological well-being.

The GAD-7 scores indicate a significant reduction in anxiety levels postintervention, with mean scores decreasing from 14.58 (moderate anxiety) to 6.67 (mild anxiety). This suggests that fat grafting in hand rejuvenation posttendon transfer has a positive psychological impact, reducing anxiety levels in patients significantly.

## Discussion

When we conducted a specific search regarding the aesthetic hand rejuvenation in cases of Hansen's disease, we found no publication to our surprise. This stimulated us to add to the existing literature on indications of autologous fat grafting in Hansens's atrophic hand.

We considered autologous fat grafting as a safe and effective technique for hand rejuvenation in patients with Hansen's disease. It addresses volume loss, improves tissue quality, and enhances hand aesthetics. The procedure involved harvesting fat from areas of excess, such as the abdomen or thighs, and injecting it into the dorsum and the palm of the hand. The fat is carefully injected in small amounts to ensure an even distribution and natural-looking results.

Our study incorporated microfat for the procedure. Microfat, compared with nanofat, does contain whole and viable adipocytes with their surrounding cell milieu. When injected, these adipocytes act like traditional fat grafts incorporating into the sites of injection. 14

Fat grafting for hand rejuvenation is a procedure developed to address the signs of aging in the hands. The appearance of our hands changes significantly as we age. The five aesthetic characteristics of youthful hands (smooth skin, firmness and elasticity, even skin tone, fullness and volume, and hydrated appearance) change as a result of both intrinsic factors (such as changes in the epidermis and dermis) and extrinsic factors. 15

Various studies in the literature show the autologous fat grafting procedure provided numerous advantages by effectively restoring lost subcutaneous fat in the hand, concealing visible veins and tendons, and reducing skin flaccidity. The literature also shows that fat grafting can lead to hand rejuvenation by promoting dermal regeneration through the presence of a higher percentage of beneficial fat cells in the subcutaneous region. 12 16 17

There are studies in which autologous fat grafting has been employed for not only aesthetic hand rejuvenation but also the treatment of patients with Raynaud's phenomenon and Dupuytren's disease, utilizing similar techniques. 18 19

Our study recorded no allergic adverse reactions. A study conducted by Fantozzi 20 also recorded no allergic reactions because the patient's own fat cells were used. There were reported adverse reactions when using synthetic fillers for hand rejuvenation. 21 However, fat cells represent a biologic filler that a plastic surgeon can safely use for tissue filling. 1 2

Fat grafting to the hand of a patient with Hansen's disease is an effective treatment that addresses issues similar to age-related volume loss with subsequent prominence of vasculature and tendons. 22 All patients exhibit volumetric augmentation, with fat retention rates on par with those observed in fat grafting procedures conducted in other anatomical sites. Subjective evaluations align with the objective metrics of transformation. 16 23 The refinement of methods has substantiated Fournier's assertion regarding the enduring nature of outcomes beyond 4 to 5 years. 4

In our study, the long-term results after the procedure proved satisfactory. The long-term results of fat grafting in Hansen's disease patients are promising, with minimal complications reported. The literature shows that fat grafting is a valuable procedure that provides long-term results. In some isolated cases, the procedure may need to be repeated. 20

We observed that edema is the most commonly encountered complication following autologous fat grafting to the hand in our study, but it typically resolves independently, as observed in many other studies. 17 19 20 24 Applying compression dressings and advising the patient to elevate their hand for a few days can help manage the swelling immediately after surgery. 7 25 Other complications that we did not encounter in our study that may resolve on their own include ecchymoses and paresthesias, as described in other studies, likely due to damage to small blood vessels and nerves. 17 20

Our study had one case with postoperative infection, which was treated with antibiotics. Studies have noted that infection is a potential complication following hand autologous fat grafting. 26 27 This issue is the primary complication that requires prevention in this procedure. To avoid infection, it is crucial to maintain sterility throughout the process. Using sterile or disposable cannulas for both fat extraction and injection is essential. Additionally, prescribing antibiotics for 1 week as a preventive measure is always recommended. 18

Our study found that physical deformities caused by Hansen's disease can have a significant psychological impact on patients, affecting their self-esteem and quality of life. Hand rejuvenation with fat grafting not only addresses aesthetic concerns but also provides psychological benefits, improving patients' self-image and confidence.

In our study, there was a significant improvement in the psychological status of the patients, assessed by the CES-D and GAD 7 scores.

This improvement helped the patients in having multiple psychological benefits.

The utilization of fat grafting has been instrumental in enhancing patients' self-esteem, thereby fostering a greater sense of confidence and ease in social interactions. This is achieved through the restoration of a more natural appearance to the hands, consequently mitigating feelings of self-consciousness and embarrassment.

Furthermore, fat grafting has proven to significantly improve both the function and aesthetic appearance of the hands, thereby contributing to an enhanced quality of life for patients by facilitating their ability to engage in daily activities.

Moreover, the application of fat grafting in hand rejuvenation has effectively mitigated social stigma and discrimination associated with Hansen's disease. By improving the aesthetic appearance of the hands, fat grafting has enabled patients to integrate more seamlessly into society.

Additionally, the psychological benefits of fat grafting cannot be understated, as evidenced by the reduction in anxiety and depression levels experienced by patients, along with an enhanced sense of body image satisfaction. This underscores the pivotal role of fat grafting in aiding physical recovery and contributing to psychological rehabilitation.

Although this procedure had been used in patients with Hansen's disease in our study, ours is primarily an aesthetic surgery unit. Therefore, we could only treat the cases that were referred to us. We did not come across any posttraumatic nerve palsy with small muscle atrophy patients with aesthetic concerns. It is also true that fat grafting in hand rejuvenation can be safely practiced in these cases as well.

## Conclusion

Fat grafting can be a valuable tool for hand rejuvenation in patients with Hansen's disease. It provides a safe and effective means of restoring hand aesthetics, providing hand filling and improving the patient's quality of life after tendon transfer surgery. Given the significant aesthetic and psychological benefits, fat grafting should be considered one of the key components in managing Hansen's disease–related hand deformities.
